# Label-Free Imaging of DNA Interactions with 2D Materials

**DOI:** 10.1021/acsphotonics.3c01604

**Published:** 2024-01-10

**Authors:** Jenny Sülzle, Wayne Yang, Yuta Shimoda, Nathan Ronceray, Eveline Mayner, Suliana Manley, Aleksandra Radenovic

**Affiliations:** † Institute of Physics and Institute of Bioengineering, Laboratory of Experimental Biophysics (LEB), École Polytechnique Fédérale de Lausanne (EPFL), Lausanne, 1015, Switzerland; ‡ Institute of Bioengineering, Laboratory of Nanoscale Biology (LBEN), École Polytechnique Fédérale de Lausanne (EPFL), Lausanne, 1015, Switzerland

**Keywords:** DNA−surface interactions, interferometric scattering, 2D materials, biosensing

## Abstract

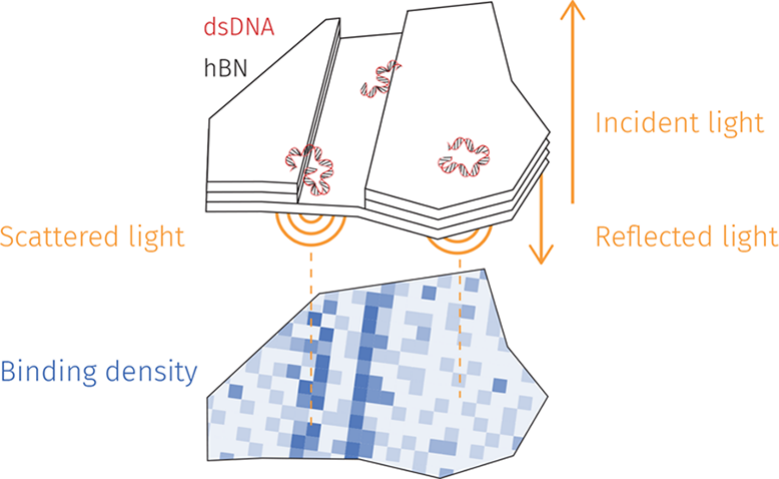

Two-dimensional (2D) materials offer potential as substrates for biosensing devices, as their properties can be engineered to tune interactions between the surface and biomolecules. Yet, not many methods can measure these interactions in a liquid environment without introducing labeling agents such as fluorophores. In this work, we harness interferometric scattering (iSCAT) microscopy, a label-free imaging technique, to investigate the interactions of single molecules of long dsDNA with 2D materials. The millisecond temporal resolution of iSCAT allows us to capture the transient interactions and to observe the dynamics of unlabeled DNA binding to a hexagonal boron nitride (hBN) surface in solution for extended periods (including a fraction of 10%, of trajectories lasting longer than 110 ms). Using a focused ion beam technique to engineer defects, we find that DNA binding affinity is enhanced at defects; when exposed to long lanes, DNA binds preferentially at the lane edges. Overall, we demonstrate that iSCAT imaging is a useful tool to study how biomolecules interact with 2D materials, a key component in engineering future biosensors.

## Introduction

Two-dimensional (2D) materials have been developed as DNA sensing platforms owing to their chemical, electronic, and mechanical properties which accommodate a variety of sensing modalities such as membrane-embedded nanopores,^1−7^ tunneling junctions,^8,9^ and field-effect transistors.^10,11^ Such devices have succeeded in sensing individual DNA strands, both optically and electronically.^12−16^ Different sensing modalities and functionalizations can be integrated to detect a whole host of biological analytes with a single device, which due to a high surface-to-volume ratio can be performed on limited sample volumes.

An important remaining challenge is to control surface interactions and to prevent fouling of the devices.^17^ Toward this end, understanding DNA interactions with 2D materials is crucial to improve biosensor designs by reducing unwanted interactions and promoting desired ones. A common approach is to label DNA fluorescently with intercalating dyes (such as YOYO-1) and study it with optical microscopy.^18^ Alternatively, atomic force microscopy (AFM) can simultaneously detect molecular binding and map the surfaces.^19,20^ Electron microscopy (EM) has also been used to image 2D materials in the presence of biomolecules at even higher spatial resolution.^21−23^ However, each of these approaches suffers from limitations. Intercalating dyes change the length and stiffness of DNA,^24,25^ and such dyes suffer from photobleaching and quenching near 2D material surfaces.^26,27^ AFM offers imaging in liquid but only for hydrophilic substrates such as mica. In the case of prototypical 2D materials, such as graphene, hexagonal boron nitride (hBN), or transition metal dichalcogenides (TMDCs), imaging is often limited to dried DNA on 2D material surfaces. EM is largely confined to dry specimens and thus lacks dynamic information or requires special liquid holders or the use of gold nanoparticles as labels.^28−32^ This may explain in part why simulation has led experiment in investigating interactions of DNA with more complex materials and device designs.^33−36^

In this work we harness interferometric scattering (iSCAT) microscopy^37,38^ to investigate single molecules of long double-stranded (ds)DNA interacting with the surface of hBN. As an analyte in solution approaches the surface, interference between the light it scatters and the light reflected by the liquid–solid interface boosts the signal contrast, enabling single-molecule detection. Because iSCAT uses the molecules themselves to generate signal, it does not require labeling. Liberated from the limited photon budget of fluorescent dyes, the technique allows millisecond temporal resolution and theoretically indefinite imaging times. So far, iSCAT has been used to study the mass distribution of short single-stranded and double-stranded DNA,^39^ protein oligomers down to molecular weights of 10 kDa,^40,41^ and the products of other biomolecular reactions^42^ as well as the dynamics of more complex cellular processes.^43^ To study the effects of surface properties on DNA binding, we study its interactions with pristine hBN as well as engineered defects and nanostructures.^44^

## Results

**Figure 1:**
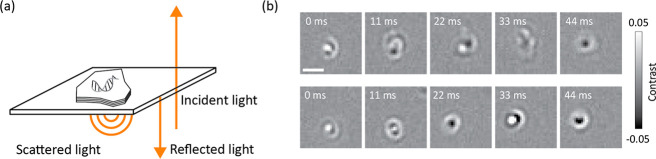
Interaction of 2D materials and DNA probed by iSCAT. (a) Schematic setup of the experiment. (b) Two examples of median-filtered time series of 20 kbp dsDNA particles diffusing on the hBN surface, illustrating changes in the shape, size, and contrast of the same molecule from frame-to-frame. Scale bar is 1 μm.

We chose hBN as our substrate due to its optical properties: it is transparent because of its band gap of 6 eV,^45^ and it uniquely among 2D materials^27,46−48^ lacks fluorescence quenching, which allows integration with fluorescent sensing strategies. Additionally, we note the potential for future engineering^33^ due to its regularly shaped crystal edges^49−51^ and single-layer films, which appear to be promising candidates for high-quality large-scale production.^52−54^ hBN was exfoliated on coverslips and imaged with iSCAT (Figure 1a, SI Experimental Section). The hBN flakes show a static uniform scattering background when illuminated in water with stronger scattering from their edges. To prevent edge scattering from overwhelming the weaker signal from dsDNA, we imaged the larger flakes and ensured they filled the selected field of view (FOV) (Figure S1). Furthermore, we use multiple frames to calculate a running averaged scattering background and produce a differential contrast image.

Upon the addition of DNA, we observed the appearance of large scattering signals (blobs) with bright and dark regions in images following median-filtered background removal (Figure 1b). We measure similar patterns for a range of different DNA lengths: 10, 20, and 48 kbp (Figure S2). These sizes were chosen based on our observed lower detection limit of 10 kbp dsDNA. We expect that the observed blobs of DNA are of single molecules, consistent with observations of both labeled and unlabeled DNA by other single-molecule sensing techniques employing similar concentrations (pM–nM) of DNA.^55−57^

Theoretical and experimental studies have demonstrated that iSCAT is highly sensitive to the axial position of scattering particles, and even nanometric changes can result in a contrast inversion in the temporally filtered images due to constructive and destructive interference.^58,59^ These studies measured biomolecules much smaller than the diffraction limit, which led to compact, circularly shaped interferometric point spread functions (iPSFs). In our case, the estimated radii of gyration for the measured DNA molecules are 238 nm (10 kbp dsDNA), 337 nm (20 kbp), and 521 nm (48 kbp). We speculate that the complex scattering signal is a superposition of multiple iPSFs from different parts of the same molecule, which span several hundred nanometers axially. Similar studies with iSCAT show a linear correlation of the contrast signal to the molecular mass of the analyte for molecules much smaller than the diffraction limit.^39,40,60^ Here, we chose the mean contrast signal of each localization to investigate its relationship to molecular weight, but we did not find a correlation for these large DNA molecules.

In time-lapse experiments, we qualitatively observe the motion of the scattering blobs (Figure 1b, Figure S3, and Movie S1). The overall signal contrast is typically low in the frames when a particle approaches or leaves the surface but increases when particles are moving along the surface. We wished to quantify this motion, but we noticed that the same molecule could change the contrast, shape, and size from frame to frame. We speculate that this is due to changing 3D conformations of the dsDNA on the hBN surface. Existing strategies for detection and localization, such as thresholding and spot detection, yield poor efficiency due to this complex appearance, which motivated us to develop a customized image analysis pipeline.

**Figure 2:**
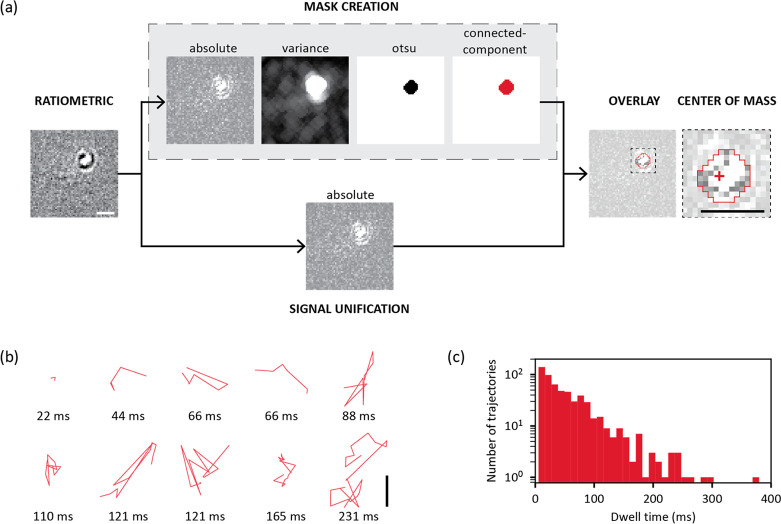
Overview of image processing workflow and trajectories on pristine hBN. (a) Schematic of image processing workflow. (b) Examples of short and long trajectories on pristine hBN. (c) Dwell time of 20 kbp DNA on pristine hBN. The histogram represents all reconstructed trajectories from three pooled measurement with each 10,000 frames imaged at 90.5 Hz. *N*(total trajectories) = 581, *N*(*L* ≤ 110 ms) = 523, *N*(*L* > 110 ms) = 58. Scale bars are 1 μm.

We developed an approach to segment and localize the particles for data with a temporal median-filtered background normalization (Figure 2a, Figures S4, S5, S6). We use the absolute value of the signal, to combine both constructive and destructive interference contributions to the signal. We optimize the threshold to avoid false positives from the noisy background, while still detecting faint signals. Each particle’s absolute contrast-weighted center of mass defines its location, which can be connected in time to reconstruct trajectories (SI Experimental Section). We manually validated the automatically tracked results by checking each step in the pipeline for a randomly selected subset of particles. We define each detected localization as a DNA binding site and the total time of reconstructed trajectories as dwell time.

On pristine hBN, we observe a wide range of trajectory geometries and lengths (Figure 2b). We split them into two groups (Figure 2c): those that are shorter than or equal to 110 ms (523 out of 581 total trajectories) and those that are longer than 110 ms (58 out of 581 total trajectories). Overall, the measured mean dwell time for 20 kbp dsDNA on pristine hBN is 51.2 [49.0, 57.4] ms (*N* = 581 trajectories, mean [95% confidence interval]). We noticed some particles diffusing out of the measured FOV (3.4 × 3.4 μm), which would lead to a slight underestimate of dwell times. The observed dynamic nature of the DNA trajectories suggests that interactions of dsDNA with the hBN surface are transient, allowing molecules to adsorb temporarily on the surface and diffuse laterally before detaching and diffusing back into the bulk solution.

Additionally, we noticed a large proportion of brief interactions; in our data set, we measured 554 single-frame binding events and 581 trajectories lasting at least two frames. Single-frame binding events likely correspond to DNA interacting transiently with the surface due to weak charge-mediated surface interactions. To test this idea, we varied the salt concentration by adding 50 mM of KCl. Under this condition, there appears to be fewer binding events per area and time (Movie S3). Transient binding events, where the DNA molecule diffuses in and out of the imaging volume versus along the surface, are expected to be short-lived, lasting less than a few milliseconds, corresponding to a fraction of a frame. Detecting these events with fluorescence microscopy would be challenging due to their brief duration; however, with iSCAT microscopy, we were able to take advantage of its temporal resolution to capture these events.

**Figure 3:**
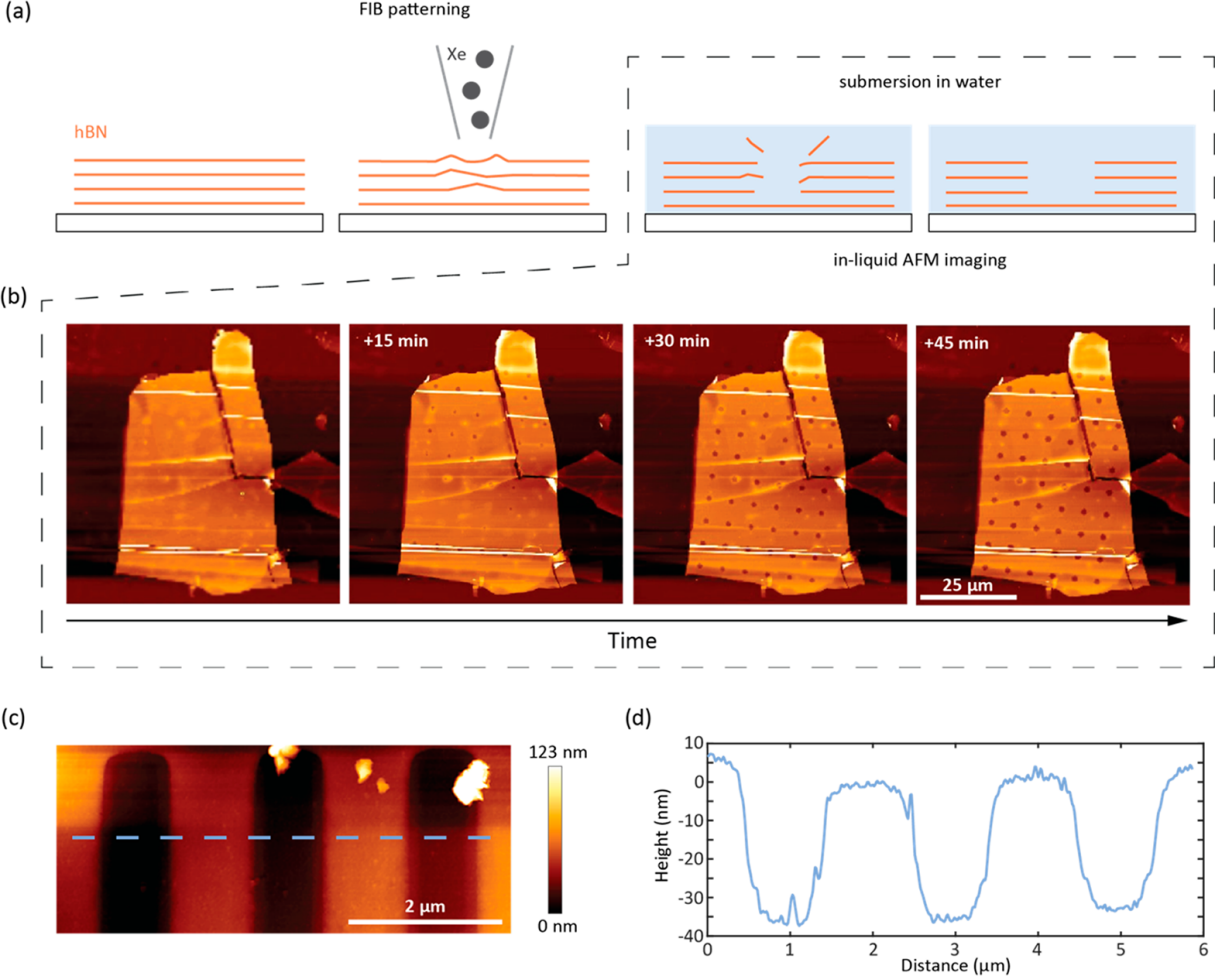
pFIB-milled, water-assisted etching of hBN. (a) Illustration of the formation of the hBN wells. (b) Time series of AFM images of a dot-patterned hBN flake upon immersion in water. (c) AFM surface map of tracks milled in an hBN flake. (d) AFM height profile scan as indicated by the dashed line in (c).

We hypothesized that it would be possible to influence the DNA binding kinetics by engineering the surface with fabricated structures that host more defects and, hence, present more binding sites. To test this idea, we turned to a recently developed surface engineering method^44^ (Figure 3a, SI Experimental Section). While the technique was first demonstrated to produce optical emitters, we saw two potential advantages for our application: there may be minimal damage to the underlying surface of hBN revealed after the water etching step is completed, and the controlled etching may reduce the background scattering of the patterned hBN surfaces and edges. As a demonstration, we patterned an array of craters and monitored the surface with AFM imaging (Figure 3b). The sample was submerged in H_2_O to etch away the patterned surface, and within 15 min, craters started forming. We continued imaging the sample until the depth of the craters stabilized at ∼12 nm after about 1 h. We also etched lanes using the same process, 1 μm wide, 35 nm deep, and spaced 1 μm apart (Figure 3c). To characterize our structures, we quantified the root mean squared (RMS) roughness of the surface. The RMS roughness along the inner trench area of the lanes is 2 nm, while for a single line scan it is even lower (0.3–0.5 nm), and that of the surrounding hBN flake area is 1.8 nm (Figure S7).

**Figure 4:**
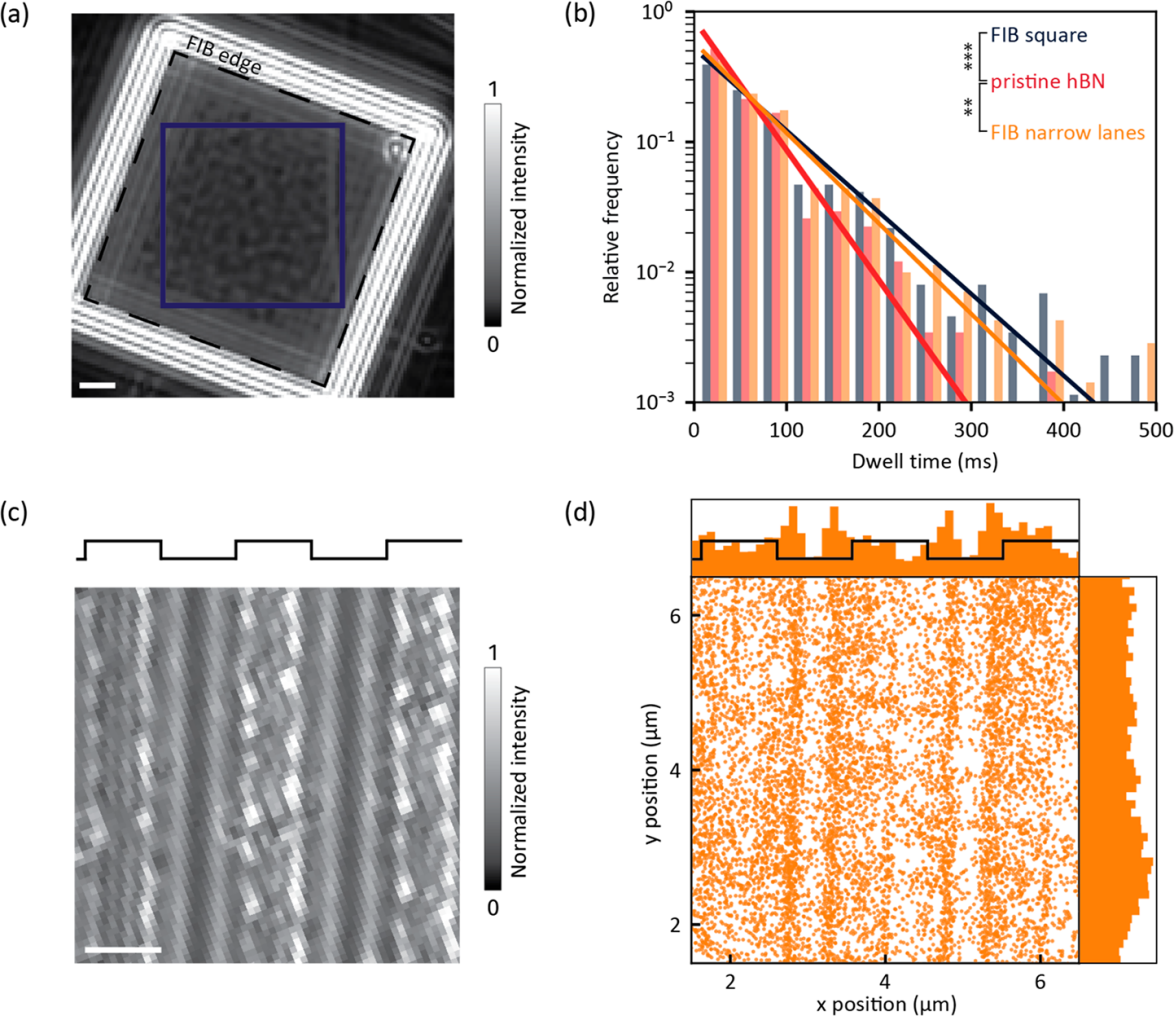
DNA interaction with FIB-milled hBN samples. (a) Native iSCAT image of an hBN flake with an etched square (dashed line). Area used for quantitative analysis is highlighted in blue. (b) Comparison of the dwell time of 20 kbp dsDNA on three different surfaces: FIB-etched hBN (FIB square), pristine hBN, and hBN patterned with lanes. The histogram represents all reconstructed trajectories from each of three pooled measurements. The data were fit to an exponentially decaying distribution. *N*(FIB square) = 874 trajectories, *N*(pristine hBN) = 581, and *N*(FIB lanes) = 703. ***P-value = 0.000021, **P-value = 0.0019. (c) Native iSCAT image of an hBN flake with etched lanes. (d) Distribution of binding spots of 20 kbp dsDNA on the sample shown in (c). The scatter histogram represents all localizations from three pooled measurements. *N* = 9130 localizations. Scale bars are 1 μm.

To observe the effect of surface modifications on the DNA binding affinity, we performed iSCAT imaging experiments on a single large square focused ion beam (FIB)-milled onto a flake. Similar FIB milling processes have yielded increased densities of optically active emitters (up to a 40× increase, Figure S8) in hBN, indicating an increase in defect sites. We reconstructed the trajectories of the DNA on the FIB-milled areas (Figure 4a) and found a mean dwell time of 77.9 [74.8, 86.4] ms (*N* = 874 trajectories, mean [95% confidence interval]; Figure 4b). This is significantly larger than the dwell time of 51.2 [49.0, 57.4] ms on the native flake. This indicates that there is an increased binding affinity, possibly from interactions with the FIB milling induced defects on the hBN flake. The nanoscopic origins and strength of these interactions are still up for debate, but a few hypotheses include increased electrostatic interactions,^36^ hydrogen bonding,^61^ stacking of the DNA on the hBN,^61,62^ and reactivity with chemical species along the defect sites.^63^

Next, we performed iSCAT imaging on hBN patterned with narrow lanes by the same process described above for the square (Figure 4c, Movie S2). The edges of the lanes offered scattering contrast, allowing us to contextualize the binding of 20 kbp of DNA to the surface. We observed an accumulation of DNA localizations along the edges of the etched lanes (*x* axis, Figure 4d, Figure S9). The peaks of the accumulation are located 200–400 nm away from the edges, within the trenches. In contrast, along the direction of the lanes (*y*-axis), binding events appear to be uniformly distributed. We examined whether scattering from the lane edges could lead to an increased contrast signal for localizations at the edges, increasing the probability of detecting events in those regions and, therefore, give a false impression of enhanced binding. Further analysis of the mean absolute contrast intensity revealed no such differences in the contrast of binding events and ruled out this possibility (Figure S10). We, therefore, interpret the data to indicate enhanced binding of DNA to the inside of the lanes. We estimate a DNA polymer radius of gyration of approximately 330 nm, consistent with the location of the peaks displaced from the edges of the lanes.

We wondered whether the preferential binding to lane edges would lead to anisotropic trajectories, e.g., DNA particles preferentially moving along the lanes. To investigate this, we created polar plots of trajectories (Figure S11) and decomposed mean squared displacement (MSD) and step size distributions, along and perpendicular to the lanes (Figures S12, S13, S14). The polar plots do not show a preferential movement along one axis, even when considering only long trajectories. Similarly, the MSD and step size distributions appeared isotropic.

## Conclusion

In summary, we present a versatile method based on iSCAT for determining dynamic behavior of DNA on two-dimensional materials, in solution and without labels. We demonstrate that iSCAT can distinguish the binding properties of the different interfaces. Additionally, the patterning of 2D material surfaces shows that it is possible to engineer the interaction with dsDNA to create sites with increased interactions. Surprisingly, we did not observe the directed motion of DNA along tracks. This calls for future exploration. Nevertheless, biosensing devices can use engineered interactions to enrich molecules within specific sensing regions^64^ such as the nanopore’s orifice,^65,66^ ultimately leading to improved sensitivity in single-molecule sensing.^67^

The data may contain more information about naked DNA’s internal configurations and dynamics. We envision that better control over the background signal and new future analyses incorporating iSCAT PSF modeling or deep-learning^41,68^ will give more insights into these aspects. Since we avoid modification to DNA’s biophysical properties arising from labeling DNA with dye,^48^ our method will help to guide the computational modeling of DNA–2D material interactions.^36,66^ Our measurements were performed with a temporal resolution of ∼11 ms, but other iSCAT instruments demonstrated a temporal resolution of up 1 MHz.^69^ This could help to investigate conformational changes in the range of microseconds.

## Data Availability

Raw images and data analysis related to the manuscript are available at https://zenodo.org/records/10361708.

## Supporting Information Available

Experimental section and figures as described in the text (PDF)
Supplementary Movie S1: Example of 20 kbp dsDNA on pristine hBN imaged with iSCAT (MP4)
Supplementary Movie S2: Example of 20 kbp dsDNA on FIB-milled hBN imaged with iSCAT (MP4)
Supplementary Movie S3: Example of 20 kbp dsDNA on pristine hBN in DI water and 50 mM KCl (MP4)

## Notes

Preprint: Jenny Sülzle, Wayne Yang, Yuta Shimoda, Nathan Ronceray, Eveline Mayner, Suliana Manley, Aleksandra Radenovic. Label-free imaging of DNA interactions with 2D materials. 2023, Biorxiv. URL 10.1101/2023.07.05.547763 (accessed December 11, 2023).

## References

[ref1] Merchant C. A.; Healy K.; Wanunu M.; Ray V.; Peterman N.; Bartel J.; Fischbein M. D.; Venta K.; Luo Z.; Johnson A. T. C.; Drndić M. DNA Translocation through Graphene Nanopores. Nano Lett. 2010, 10 (8), 2915–2921. 10.1021/nl101046t. 20698604

[ref2] Garaj S.; Hubbard W.; Reina A.; Kong J.; Branton D.; Golovchenko J. A. Graphene as a Subnanometre Trans-Electrode Membrane. Nature 2010, 467 (7312), 190–193. 10.1038/nature09379. 20720538 PMC2956266

[ref3] Verschueren D. V.; Yang W.; Dekker C. Lithography-Based Fabrication of Nanopore Arrays in Freestanding SiN and Graphene Membranes. Nanotechnology 2018, 29 (14), 145302 10.1088/1361-6528/aaabce. 29384130 PMC5997186

[ref4] Graf M.; Lihter M.; Thakur M.; Georgiou V.; Topolancik J.; Ilic B. R.; Liu K.; Feng J.; Astier Y.; Radenovic A. Fabrication and Practical Applications of Molybdenum Disulfide Nanopores. Nat. Protoc. 2019, 14 (4), 1130–1168. 10.1038/s41596-019-0131-0. 30903110

[ref5] Schneider G. F.; Kowalczyk S. W.; Calado V. E.; Pandraud G.; Zandbergen H. W.; Vandersypen L. M. K.; Dekker C. DNA Translocation through Graphene Nanopores. Nano Lett. 2010, 10 (8), 3163–3167. 10.1021/nl102069z. 20608744

[ref6] Liu K.; Pan C.; Kuhn A.; Nievergelt A. P.; Fantner G. E.; Milenkovic O.; Radenovic A. Detecting Topological Variations of DNA at Single-Molecule Level. Nat. Commun. 2019, 10 (1), 3 10.1038/s41467-018-07924-1. 30602774 PMC6315031

[ref7] Danda G.; Masih Das P.; Chou Y.-C.; Mlack J. T.; Parkin W. M.; Naylor C. H.; Fujisawa K.; Zhang T.; Fulton L. B.; Terrones M.; Johnson A. T. C.; Drndić M. Monolayer WS2 Nanopores for DNA Translocation with Light-Adjustable Sizes. ACS Nano 2017, 11 (2), 1937–1945. 10.1021/acsnano.6b08028. 28125779 PMC5893941

[ref8] Caneva S.; Gehring P.; García-Suárez V. M.; García-Fuente A.; Stefani D.; Olavarria-Contreras I. J.; Ferrer J.; Dekker C.; van der Zant H. S. J. Mechanically Controlled Quantum Interference in Graphene Break Junctions. Nat. Nanotechnol. 2018, 13 (12), 1126–1131. 10.1038/s41565-018-0258-0. 30224794

[ref9] Huang S.; He J.; Chang S.; Zhang P.; Liang F.; Li S.; Tuchband M.; Fuhrmann A.; Ros R.; Lindsay S. Identifying Single Bases in a DNA Oligomer with Electron Tunnelling. Nat. Nanotechnol. 2010, 5 (12), 868–873. 10.1038/nnano.2010.213. 21076404 PMC4121130

[ref10] Heerema S. J.; Dekker C. Graphene Nanodevices for DNA Sequencing. Nat. Nanotechnol. 2016, 11 (2), 127–136. 10.1038/nnano.2015.307. 26839258

[ref11] Girdhar A.; Sathe C.; Schulten K.; Leburton J.-P. Graphene Quantum Point Contact Transistor for DNA Sensing. Proc. Natl. Acad. Sci. U. S. A. 2013, 110 (42), 16748–16753. 10.1073/pnas.1308885110. 24082108 PMC3801026

[ref12] Yamazaki H.; Kimura S.; Tsukahara M.; Esashika K.; Saiki T. Optical Detection of DNA Translocation through Silicon Nanopore by Ultraviolet Light. Appl. Phys. A: Mater. Sci. Process. 2014, 115 (1), 53–56. 10.1007/s00339-013-7956-0.

[ref13] Soni G. V.; Singer A.; Yu Z.; Sun Y.; McNally B.; Meller A. Synchronous Optical and Electrical Detection of Biomolecules Traversing through Solid-State Nanopores. Rev. Sci. Instrum. 2010, 81 (1), 014301 10.1063/1.3277116. 20113116 PMC2821415

[ref14] Assad O. N.; Di Fiori N.; Squires A. H.; Meller A. Two Color DNA Barcode Detection in Photoluminescence Suppressed Silicon Nitride Nanopores. Nano Lett. 2015, 15 (1), 745–752. 10.1021/nl504459c. 25522780 PMC4296929

[ref15] Yang W.; Dekker C. Single-Molecule Ionic and Optical Sensing with Nanoapertures. In Single Molecule Sensing Beyond Fluorescence; Bowen W.; Vollmer F.; Gordon R., Eds.; Nanostructure Science and Technology; Springer International Publishing: Cham, 2022; pp 367–387.

[ref16] Pitchford W. H.; Kim H.-J.; Ivanov A. P.; Kim H.-M.; Yu J.-S.; Leatherbarrow R. J.; Albrecht T.; Kim K.-B.; Edel J. B. Synchronized Optical and Electronic Detection of Biomolecules Using a Low Noise Nanopore Platform. ACS Nano 2015, 9 (2), 1740–1748. 10.1021/nn506572r. 25635821

[ref17] Schneider G. F.; Xu Q.; Hage S.; Luik S.; Spoor J. N. H.; Malladi S.; Zandbergen H.; Dekker C. Tailoring the Hydrophobicity of Graphene for Its Use as Nanopores for DNA Translocation. Nat. Commun. 2013, 4 (1), 2619 10.1038/ncomms3619. 24126320

[ref18] Lu C.; Liu Y.; Ying Y.; Liu J. Comparison of MoS2, WS2, and Graphene Oxide for DNA Adsorption and Sensing. Langmuir 2017, 33 (2), 630–637. 10.1021/acs.langmuir.6b04502. 28025885

[ref19] Pyne A.; Thompson R.; Leung C.; Roy D.; Hoogenboom B. W. Single-Molecule Reconstruction of Oligonucleotide Secondary Structure by Atomic Force Microscopy. Small 2014, 10 (16), 3257–3261. 10.1002/smll.201400265. 24740866

[ref20] Pyne A. L. B.; Noy A.; Main K. H. S.; Velasco-Berrelleza V.; Piperakis M. M.; Mitchenall L. A.; Cugliandolo F. M.; Beton J. G.; Stevenson C. E. M.; Hoogenboom B. W.; Bates A. D.; Maxwell A.; Harris S. A. Base-Pair Resolution Analysis of the Effect of Supercoiling on DNA Flexibility and Major Groove Recognition by Triplex-Forming Oligonucleotides. Nat. Commun. 2021, 12 (1), 1053 10.1038/s41467-021-21243-y. 33594049 PMC7887228

[ref21] Gentile F.; Moretti M.; Limongi T.; Falqui A.; Bertoni G.; Scarpellini A.; Santoriello S.; Maragliano L.; Proietti Zaccaria R.; di Fabrizio E. Direct Imaging of DNA Fibers: The Visage of Double Helix. Nano Lett. 2012, 12 (12), 6453–6458. 10.1021/nl3039162. 23171353

[ref22] Zellweger R.; Lopes M. Dynamic Architecture of Eukaryotic DNA Replication Forks In Vivo, Visualized by Electron Microscopy. In Genome Instability: Methods and Protocols; Muzi-Falconi M.; Brown G. W., Eds.; Methods in Molecular Biology; Springer: New York, NY, 2018; pp 261–294. 10.1007/978-1-4939-7306-4_19 29043630

[ref23] Cerf A.; Alava T.; Barton R. A.; Craighead H. G. Transfer-Printing of Single DNA Molecule Arrays on Graphene for High-Resolution Electron Imaging and Analysis. Nano Lett. 2011, 11 (10), 4232–4238. 10.1021/nl202219w. 21919532 PMC3205448

[ref24] Günther K.; Mertig M.; Seidel R. Mechanical and Structural Properties of YOYO-1 Complexed DNA. Nucleic Acids Res. 2010, 38 (19), 6526–6532. 10.1093/nar/gkq434. 20511588 PMC2965214

[ref25] Rocha M. S.; Ferreira M. C.; Mesquita O. N. Transition on the Entropic Elasticity of DNA Induced by Intercalating Molecules. J. Chem. Phys. 2007, 127 (10), 105108 10.1063/1.2768945. 17867787

[ref26] Tan A. T. L.; Kim J.; Huang J.-K.; Li L.-J.; Huang J. Seeing Two-Dimensional Sheets on Arbitrary Substrates by Fluorescence Quenching Microscopy. Small 2013, 9 (19), 3253–3258. 10.1002/smll.201300049. 23554324

[ref27] Kasry A.; Ardakani A. A.; Tulevski G. S.; Menges B.; Copel M.; Vyklicky L. Highly Efficient Fluorescence Quenching with Graphene. J. Phys. Chem. C 2012, 116 (4), 2858–2862. 10.1021/jp207972f.

[ref28] Dunn G.; Adiga V. P.; Pham T.; Bryant C.; Horton-Bailey D. J.; Belling J. N.; LaFrance B.; Jackson J. A.; Barzegar H. R.; Yuk J. M.; Aloni S.; Crommie M. F.; Zettl A. Graphene-Sealed Flow Cells for In Situ Transmission Electron Microscopy of Liquid Samples. ACS Nano 2020, 14 (8), 9637–9643. 10.1021/acsnano.0c00431. 32806056

[ref29] Yuk J. M.; Park J.; Ercius P.; Kim K.; Hellebusch D. J.; Crommie M. F.; Lee J. Y.; Zettl A.; Alivisatos A. P. High-Resolution EM of Colloidal Nanocrystal Growth Using Graphene Liquid Cells. Science 2012, 336 (6077), 61–64. 10.1126/science.1217654. 22491849

[ref30] de Jonge N.; Ross F. M. Electron Microscopy of Specimens in Liquid. Nat. Nanotechnol. 2011, 6 (11), 695–704. 10.1038/nnano.2011.161. 22020120

[ref31] Zheng H.; Claridge S. A.; Minor A. M.; Alivisatos A. P.; Dahmen U. Nanocrystal Diffusion in a Liquid Thin Film Observed by in Situ Transmission Electron Microscopy. Nano Lett. 2009, 9 (6), 2460–2465. 10.1021/nl9012369. 19408927

[ref32] Chen Q.; Smith J. M.; Park J.; Kim K.; Ho D.; Rasool H. I.; Zettl A.; Alivisatos A. P. 3D Motion of DNA-Au Nanoconjugates in Graphene Liquid Cell Electron Microscopy. Nano Lett. 2013, 13 (9), 4556–4561. 10.1021/nl402694n. 23944844

[ref33] Zhang L.; Wang X. DNA Sequencing by Hexagonal Boron Nitride Nanopore: A Computational Study. Nanomaterials 2016, 6 (6), 111 10.3390/nano6060111. 28335237 PMC5302631

[ref34] Manzanares-Palenzuela C. L.; Pourrahimi A. M.; Gonzalez-Julian J.; Sofer Z.; Pykal M.; Otyepka M.; Pumera M. Interaction of Single- and Double-Stranded DNA with Multilayer MXene by Fluorescence Spectroscopy and Molecular Dynamics Simulations. Chem. Sci. 2019, 10 (43), 10010–10017. 10.1039/C9SC03049B. 32055358 PMC6979399

[ref35] Chen S. H.; Bell D. R.; Luan B. Understanding Interactions between Biomolecules and Two-Dimensional Nanomaterials Using in Silico Microscopes. Adv. Drug Delivery Rev. 2022, 186, 114336 10.1016/j.addr.2022.114336. PMC9212071 35597306

[ref36] Shankla M.; Aksimentiev A. Step-Defect Guided Delivery of DNA to a Graphene Nanopore. Nat. Nanotechnol. 2019, 14 (9), 858–865. 10.1038/s41565-019-0514-y. 31384038 PMC6863603

[ref37] Lindfors K.; Kalkbrenner T.; Stoller P.; Sandoghdar V. Detection and Spectroscopy of Gold Nanoparticles Using Supercontinuum White Light Confocal Microscopy. Phys. Rev. Lett. 2004, 93 (3), 037401 10.1103/PhysRevLett.93.037401. 15323866

[ref38] Jacobsen V.; Stoller P.; Brunner C.; Vogel V.; Sandoghdar V. Interferometric Optical Detection and Tracking of Very Small Gold Nanoparticles at a Water-Glass Interface. Opt. Express 2006, 14 (1), 405 10.1364/OPEX.14.000405. 19503354

[ref39] Li Y.; Struwe W. B.; Kukura P. Single Molecule Mass Photometry of Nucleic Acids. Nucleic Acids Res. 2020, 48 (17), e97 10.1093/nar/gkaa632. 32756898 PMC7515692

[ref40] Young G.; Hundt N.; Cole D.; Fineberg A.; Andrecka J.; Tyler A.; Olerinyova A.; Ansari A.; Marklund E. G.; Collier M. P.; Chandler S. A.; Tkachenko O.; Allen J.; Crispin M.; Billington N.; Takagi Y.; Sellers J. R.; Eichmann C.; Selenko P.; Frey L.; Riek R.; Galpin M. R.; Struwe W. B.; Benesch J. L. P.; Kukura P. Quantitative Mass Imaging of Single Biological Macromolecules. Science 2018, 360 (6387), 423–427. 10.1126/science.aar5839. 29700264 PMC6103225

[ref41] Dahmardeh M.; Mirzaalian Dastjerdi H.; Mazal H.; Köstler H.; Sandoghdar V. Self-Supervised Machine Learning Pushes the Sensitivity Limit in Label-Free Detection of Single Proteins below 10 kDa. Nat. Methods 2023, 20 (3), 442–447. 10.1038/s41592-023-01778-2. 36849549 PMC9998267

[ref42] Hundt N.; Cole D.; Hantke M. F.; Miller J. J.; Struwe W. B.; Kukura P. Direct Observation of the Molecular Mechanism Underlying Protein Polymerization. Science Advances 2022, 8 (35), eabm7935 10.1126/sciadv.abm7935. 36044567 PMC9432825

[ref43] Küppers M.; Albrecht D.; Kashkanova A. D.; Lühr J.; Sandoghdar V. Confocal Interferometric Scattering Microscopy Reveals 3D Nanoscopic Structure and Dynamics in Live Cells. Nat. Commun. 2023, 14 (1), 1962 10.1038/s41467-023-37497-7. 37029107 PMC10081331

[ref44] Glushkov E.; Macha M.; Räth E.; Navikas V.; Ronceray N.; Cheon C. Y.; Ahmed A.; Avsar A.; Watanabe K.; Taniguchi T.; Shorubalko I.; Kis A.; Fantner G.; Radenovic A. Engineering Optically Active Defects in Hexagonal Boron Nitride Using Focused Ion Beam and Water. ACS Nano 2022, 16 (3), 3695–3703. 10.1021/acsnano.1c07086. 35254820 PMC8945698

[ref45] Wickramaratne D.; Weston L.; Van de Walle C. G. Monolayer to Bulk Properties of Hexagonal Boron Nitride. J. Phys. Chem. C 2018, 122 (44), 25524–25529. 10.1021/acs.jpcc.8b09087.

[ref46] Deng H.; Yang X.; Gao Z. MoS2 Nanosheets as an Effective Fluorescence Quencher for DNA Methyltransferase Activity Detection. Analyst 2015, 140 (9), 3210–3215. 10.1039/C4AN02133A. 25760806

[ref47] Kaminska I.; Bohlen J.; Rocchetti S.; Selbach F.; Acuna G. P.; Tinnefeld P. Distance Dependence of Single-Molecule Energy Transfer to Graphene Measured with DNA Origami Nanopositioners. Nano Lett. 2019, 19 (7), 4257–4262. 10.1021/acs.nanolett.9b00172. 31251640

[ref48] Richter L.; Szalai A. M.; Manzanares-Palenzuela C. L.; Kamińska I.; Tinnefeld P. Exploring the Synergies of Single-Molecule Fluorescence and 2D Materials Coupled by DNA. Adv. Mater. 2023, 35 (41), 2303152 10.1002/adma.202303152. 37670535

[ref49] Liu K.; Lihter M.; Sarathy A.; Caneva S.; Qiu H.; Deiana D.; Tileli V.; Alexander D. T. L.; Hofmann S.; Dumcenco D.; Kis A.; Leburton J.-P.; Radenovic A. Geometrical Effect in 2D Nanopores. Nano Lett. 2017, 17 (7), 4223–4230. 10.1021/acs.nanolett.7b01091. 28592108

[ref50] Dai C.; Popple D.; Su C.; Park J.-H.; Watanabe K.; Taniguchi T.; Kong J.; Zettl A. Evolution of Nanopores in Hexagonal Boron Nitride. Commun. Chem. 2023, 6 (1), 108 10.1038/s42004-023-00899-1. 37277463 PMC10241886

[ref51] Gilbert S. M.; Dunn G.; Azizi A.; Pham T.; Shevitski B.; Dimitrov E.; Liu S.; Aloni S.; Zettl A. Fabrication of Subnanometer-Precision Nanopores in Hexagonal Boron Nitride. Sci. Rep. 2017, 7 (1), 15096 10.1038/s41598-017-12684-x. 29118413 PMC5678191

[ref52] Caneva S.; Weatherup R. S.; Bayer B. C.; Brennan B.; Spencer S. J.; Mingard K.; Cabrero-Vilatela A.; Baehtz C.; Pollard A. J.; Hofmann S. Nucleation Control for Large, Single Crystalline Domains of Monolayer Hexagonal Boron Nitride via Si-Doped Fe Catalysts. Nano Lett. 2015, 15 (3), 1867–1875. 10.1021/nl5046632. 25664483 PMC4358078

[ref53] Fukamachi S.; Solís-Fernández P.; Kawahara K.; Tanaka D.; Otake T.; Lin Y.-C.; Suenaga K.; Ago H. Large-Area Synthesis and Transfer of Multilayer Hexagonal Boron Nitride for Enhanced Graphene Device Arrays. Nat. Electron. 2023, 6 (2), 126–136. 10.1038/s41928-022-00911-x.

[ref54] Liu S.; He R.; Ye Z.; Du X.; Lin J.; Jiang H.; Liu B.; Edgar J. H. Large-Scale Growth of High-Quality Hexagonal Boron Nitride Crystals at Atmospheric Pressure from an Fe-Cr Flux. Cryst. Growth Des. 2017, 17 (9), 4932–4935. 10.1021/acs.cgd.7b00871.

[ref55] Bell N. A. W.; Muthukumar M.; Keyser U. F. Translocation Frequency of Double-Stranded DNA through a Solid-State Nanopore. Phys. Rev. E 2016, 93 (2), 022401 10.1103/PhysRevE.93.022401. 26986356 PMC4985240

[ref56] Wanunu M.; Morrison W.; Rabin Y.; Grosberg A. Y.; Meller A. Electrostatic Focusing of Unlabelled DNA into Nanoscale Pores Using a Salt Gradient. Nat. Nanotechnol. 2010, 5 (2), 160–165. 10.1038/nnano.2009.379. 20023645 PMC2849735

[ref57] Lastra L. S.; Bandara Y. M. N. D. Y.; Nguyen M.; Farajpour N.; Freedman K. J. On the Origins of Conductive Pulse Sensing inside a Nanopore. Nat. Commun. 2022, 13 (1), 2186 10.1038/s41467-022-29758-8. 35562332 PMC9106702

[ref58] Mahmoodabadi R. G.; Taylor R. W.; Kaller M.; Spindler S.; Mazaheri M.; Kasaian K.; Sandoghdar V. Point Spread Function in Interferometric Scattering Microscopy (iSCAT). Part I: Aberrations in Defocusing and Axial Localization. Opt. Express 2020, 28 (18), 25969–25988. 10.1364/OE.401374. 32906875

[ref59] Dong J.; Maestre D.; Conrad-Billroth C.; Juffmann T. Fundamental Bounds on the Precision of iSCAT, COBRI and Dark-Field Microscopy for 3D Localization and Mass Photometry. J. Phys. D: Appl. Phys. 2021, 54 (39), 394002 10.1088/1361-6463/ac0f22.

[ref60] Heermann T.; Steiert F.; Ramm B.; Hundt N.; Schwille P. Mass-Sensitive Particle Tracking to Elucidate the Membrane-Associated MinDE Reaction Cycle. Nat. Methods 2021, 18 (10), 1239–1246. 10.1038/s41592-021-01260-x. 34608318 PMC8490154

[ref61] Ding N.; Chen X.; Wu C.-M. L.; Li H. Adsorption of Nucleobase Pairs on Hexagonal Boron Nitride Sheet: Hydrogen Bonding versus Stacking. Phys. Chem. Chem. Phys. 2013, 15 (26), 10767–10776. 10.1039/c3cp50912e. 23689542

[ref62] Lee J.-H.; Choi Y.-K.; Kim H.-J.; Scheicher R. H.; Cho J.-H. Physisorption of DNA Nucleobases on H-BN and Graphene: vdW-Corrected DFT Calculations. J. Phys. Chem. C 2013, 117 (26), 13435–13441. 10.1021/jp402403f.

[ref63] Ronceray N.; You Y.; Glushkov E.; Lihter M.; Rehl B.; Chen T.-H.; Nam G.-H.; Borza F.; Watanabe K.; Taniguchi T.; Roke S.; Keerthi A.; Comtet J.; Radha B.; Radenovic A. Liquid-Activated Quantum Emission from Pristine Hexagonal Boron Nitride for Nanofluidic Sensing. Nat. Mater. 2023, 22 (10), 1236–1242. 10.1038/s41563-023-01658-2. 37652991 PMC10533396

[ref64] Shin D. H.; Kim K.; Costic K.; Watanabe K.; Taniguchi T.; Gerard V.; Sabina C.; Aleksei A.; Peter G. S.; Chirlmin J. Diffusion of DNA on Atomically Flat 2D Material Surfaces. bioRxiv 2023, 2023.11.01.565159 10.1101/2023.11.01.565159.

[ref65] Cao Z.; Yadav P.; Barati Farimani A. Which 2D Material Is Better for DNA Detection: Graphene, MoS2, or MXene?. Nano Lett. 2022, 22 (19), 7874–7881. 10.1021/acs.nanolett.2c02603. 36165777

[ref66] Wells D. B.; Belkin M.; Comer J.; Aksimentiev A. Assessing Graphene Nanopores for Sequencing DNA. Nano Lett. 2012, 12 (8), 4117–4123. 10.1021/nl301655d. 22780094 PMC3434709

[ref67] Shin D. H.; Yang X.; Caneva S. Single-Molecule Protein Fingerprinting with Photonic Hexagonal Boron Nitride Nanopores. Acc. Mater. Res. 2023, 4 (4), 307–310. 10.1021/accountsmr.3c00016. 37151913 PMC10152444

[ref68] Boyle M. J.; Goldman Y. E.; Composto R. J. Enhancing Nanoparticle Detection in Interferometric Scattering (iSCAT) Microscopy Using a Mask R-CNN. J. Phys. Chem. B 2023, 127 (16), 3737–3745. 10.1021/acs.jpcb.3c00097. 37074024 PMC11966666

[ref69] Spindler S.; Ehrig J.; König K.; Nowak T.; Piliarik M.; Stein H. E.; Taylor R. W.; Garanger E.; Lecommandoux S.; Alves I. D.; Sandoghdar V. Visualization of Lipids and Proteins at High Spatial and Temporal Resolution via Interferometric Scattering (iSCAT) Microscopy. J. Phys. D: Appl. Phys. 2016, 49 (27), 274002 10.1088/0022-3727/49/27/274002.

